# The Association of* Streptococcus gallolyticus* Subspecies* pasteurianus* Bacteremia with the Detection of Premalignant and Malignant Colonic Lesions

**DOI:** 10.1155/2016/7815843

**Published:** 2016-07-31

**Authors:** Gaurav Chand, Leonid Shamban, Adam Forman, Prabhat Sinha

**Affiliations:** ^1^Department of Internal Medicine, Providence Hospital and Medical Center, 16001 W. Nine Mile Road, Southfield, MI 48075, USA; ^2^Department of Gastroenterology, Genesys Regional Medical Center, One Genesys Parkway, Grand Blanc, MI 48439, USA; ^3^Department of Hematology and Oncology, Providence Hospital and Medical Center, 16001 W. Nile Mile Road, Southfield, MI 48075, USA; ^4^Department of Pulmonology and Critical Care, Providence Hospital and Medical Center, 16001 W. Nine Mile Road, Southfield, MI 48075, USA

## Abstract

*Streptococcus gallolyticus* subspecies (subsp.)* gallolyticus* (formerly* bovis* biotype I) bacteremia has been associated with colonic adenocarcinoma. The* bovis* species underwent reclassification in 2003. Subtypes of* gallolyticus* are associated with colonic malignancy but are less frequent, resulting in less awareness. A 71-year-old male admitted with worsening lower back pain and fevers. Initial vital signs and laboratory data were within normal limits. MRI revealed lumbosacral osteomyelitis and antibiotics were initiated. Blood cultures showed* Streptococcus* species, prompting a transesophageal echocardiogram (TEE) revealing vegetations on the mitral and aortic valves. The etiology for his endocarditis was unclear. A colonoscopy was suggested, but his clinical instability made such a procedure intolerable. Final cultures revealed* Streptococcus gallolyticus* subsp.* pasteurianus* (previously* bovis* biotype II). After antibiotic completion he underwent aortic grafting with valve replacements. Later, he was readmitted for* Streptococcus* bacteremia. After a negative TEE, colonoscopy revealed a 2.5 × 3 cm cecal tubulovillous adenoma with high-grade dysplasia suspicious for his origin of infection. Clinicians understand the link between* Streptococcus gallolyticus* subsp.* gallolyticus* (*bovis* type I) and malignancy, but the new speciation may be unfamiliar. There are no guidelines for managing* S. gallolyticus* subsp.* pasteurianus* bacteremia; therefore a colonoscopy should be considered when no source is identified.

## 1. Introduction

*Streptococcus bovis* biotype I bacteremia has been long associated with infective endocarditis due to an underlying colonic malignancy since its first documented case in 1951 [1]. Previous reports have suggested that* Streptococcus bovis* type I bacteremia has been implicated with concomitant colonic tumors in 25 to 80% of patients [1], as well as the presence of premalignant colonic lesions [2–4]. In the late 1990s and early 2000s, the* Streptococcus bovis* species underwent significant taxonomic changes that resulted in the renaming of* S*.* bovis* biotype I as* Streptococcus gallolyticus* subsp.* gallolyticus*, biotype II/1 as* S. lutetiensis* (i.e.,* S. infantarius* subsp.* infantarius*), and biotype II/2 as* S. gallolyticus* subsp.* pasteurianus* [3, 4]. Multiple reports show that* Streptococcus gallolyticus* subtypes are also associated with colonic malignancy; however, they are less frequent compared to* Streptococcus gallolyticus* subsp.* gallolyticus* [5, 6]. Years later, many clinicians continue to be unfamiliar with the new speciation [7]. Therefore, these organism findings in clinical practice must be highlighted to make clinicians more comfortable with their management. Herein, we report a rarely associated entity of* S. gallolyticus* subsp.* pasteurianus* bacteremia and infective endocarditis with subsequent detection of a tubulovillous adenoma with high-grade dysplasia.

## 2. Case Report

A 70-year-old Caucasian male presented to the hospital with complaints of progressively worsening low back pain radiating into his bilateral hips ongoing for the past six weeks. Initially, the patient saw a physician in the outpatient setting who evaluated his back pain with a magnetic resonance imaging test (MRI) that revealed degenerative disc disease at the L_5_-S_1_ level. He was prompted to come to the hospital as his pain significantly worsened over the past ten days resulting in the inability to ambulate. He denied any symptoms of neurological impairment. Additionally, he was noted to be experiencing fevers and chills for the past few weeks, which he attributed to his history of chronic prostatitis.

His past medical history is remarkable for hypertension, hyperlipidemia, chronic prostatitis, and benign prostatic hypertrophy. The patient is retired from the automotive business, lives with his wife, and reported no use of tobacco, alcohol, or illicit drugs. He reported no recent travel. His initial vital signs yielded a blood pressure of 118/74 mmHg, heart rate of 135 beats per minute, respiratory rate of 17 breaths per minute, temperature of 98.5° Fahrenheit (F), and an oxygen saturation of 96 percent on room air. Physical exam revealed a well-nourished male, who was alert and oriented to person, place, time, and situation. Cranial nerves II–XII were grossly intact bilaterally with +5/5 gross bilateral upper extremity strength and +4/5 gross bilateral lower extremity strength. Sensation to noxious and tactile stimuli was intact. Deep tendon reflexes were equal and +1/4 in bilateral lower extremities. The patient experienced significant back pain during the examination, while the rest of his physical exam was unremarkable including his cardiac exam.

Complete blood count results included a white blood cell count, 11.2 K/*μ*L; hemoglobin, 13.1 g/dL; hematocrit, 38.7%; and platelets, 144 K/*μ*L. Chemistry panel showed sodium, 133 mmol/L; potassium, 4.1 mmol/L; chloride, 98 mmol/L; CO_2_, 24 mmol/L; blood urea nitrogen, 22 mg/dL; and creatinine, 0.9 mg/dL. An initial computed tomography scan (CT scan) without contrast of the abdomen and pelvis showed nonobstructing renal stones, and a magnetic resonance imaging study (MRI) of the lumbosacral spine showed changes consistent with L_3_-L_4_ and L_5_-S_1_ osteomyelitis. Blood cultures were drawn, and the patient was admitted with the diagnosis of lumbosacral osteomyelitis. He was started on broad-spectrum antibiotics. The following day, preliminary blood cultures revealed beta-hemolytic* Streptococcus* bacteremia in two out of two sets, and antibiotics were adjusted to intravenous vancomycin and gentamycin.

During the hospital course, the patient continued to complain of weakness, fatigue, and some chest discomfort despite intravenous antibiotic therapy. A transthoracic echocardiogram exhibited vegetations on mitral and aortic valves, mildly dilated aorta, and a moderate-sized mass on the noncoronary cusp measuring 1.2 by 1.0 centimeters with preserved ejection fraction. These findings prompted a transesophageal echocardiogram that revealed an aortic wall abscess with a mobile tip and vegetations over the mitral valve accompanied by severe mitral valve regurgitation and aortic valve incompetence. Final blood cultures were consistent with* Streptococcus gallolyticus* subsp.* pasteurianus*, and antibiotic therapy was tailored to intravenous ceftriaxone and gentamycin. Due to this discovery, a concern for a colonic etiology arose, as this species is a variant of the previous* Streptococcus bovis* species. However, these results could not be further investigated due the patient's clinical status.

The patient developed problems with his neck mobility that prompted a CT scan that revealed cervical spine discitis at C_5_-C_6_ level, which was later confirmed by MRI. Subsequently, the patient underwent three operations over the course of the next two weeks. Neurosurgery had operated on the patient's cervical and lumbosacral discitis in two separate surgeries. Cardiothoracic surgery replaced the patient's mitral and aortic valves and placed an ascending aortic graft due to an aneurysm. Following these surgical procedures and stabilization, the patient was discharged to a subacute rehabilitation facility with a peripherally inserted central catheter for twelve weeks of intravenous ceftriaxone infusion.

**Figure 1:**
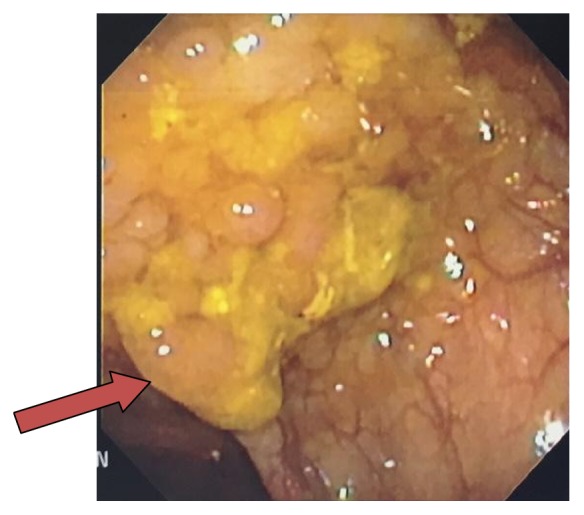
Colonoscopy. Colonoscopy findings revealing a 2.5 × 1.0 cm cecal mass.

After undergoing months of rehabilitation, the patient was readmitted five months from his initial presentation for pyrexia of 100.4°F. Blood cultures were obtained and revealed two out of two sets consistent with gram positive cocci in clusters resembling* Streptococcus*. The patient was reevaluated for osteomyelitis and MRI of the cervical, thoracic, and lumbar spine revealed no acute infection. An endocarditis workup was also unremarkable. The final report for blood cultures showed* Lactococcus garvieae*, and an evaluation for a colonoscopy was undertaken, since there have been few case reports relating colonic pathology as the source of the bacteremia [8, 9]. The patient was advised to have a colonoscopy in the outpatient setting after being discharged from the rehab center, but he did not comply. Colonoscopy revealed a 2.5 by 3.0 centimeter cecal mass (Figure 1), two sessile descending colonic polyps, and mild internal hemorrhoids.

**Figure 2:**
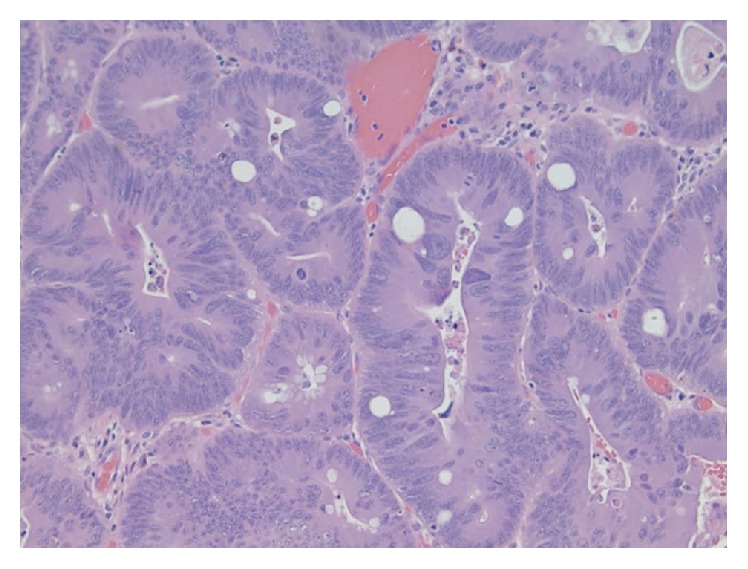
Pathology slide. Pathology exhibiting a tubulovillous adenoma with high-grade dysplasia shown under H&E staining at 40x magnification.

Final pathology yielded a tubulovillous adenoma in the cecum (Figure 2) and hyperplastic polyps in the descending colon.

At this time, the patient was discharged with intravenous vancomycin for three weeks. After completing antibiotic therapy, the patient underwent a right-sided hemicolectomy, and the final surgical pathology showed a tubulovillous adenoma with high-grade dysplasia. Following stabilization during the postoperative period, the patient was discharged in fair condition to a subacute rehabilitation facility.

## 3. Discussion

In the preceding case, our patient exhibited profound recurrent systemic bacteremia that was eventually attributed to a colonic source. Many clinicians understand the link between* Streptococcus gallolyticus* subsp.* gallolyticus* (*bovis* type I) and malignancy, but the new speciation may be unfamiliar to practitioners.* S. gallolyticus* subsp.* gallolyticus* has a much greater association than* S. gallolyticus* subsp.* pasteurianus* with colonic malignancies as is depicted in Table 1 [10].

In a literature review, the association of* S*.* gallolyticus* subsp.* gallolyticus* bacteremia was 94% and 71% with clinical infectious endocarditis and underlying colonic malignancy, respectively [11]. However,* S*.* gallolyticus* subsp.* pasteurianus* bacteremia is less commonly associated with infective endocarditis and occult colonic malignancy at rates of 18% and 17%, respectively [11]. Regardless of the weaker association of* S*.* gallolyticus* subsp.* pasteurianus* with underlying colonic pathology, we strongly believe that patients exhibiting any* S*.* bovis* bacteremia (type I or II) should undergo a screening colonoscopy. It has also been noted in previous studies that* S*.* bovis* is also associated with premalignant colonic lesions, which are often asymptomatic and undetectable by fecal occult blood testing [12].

Interestingly, confounding in this case is the readmission of bacteremia with a different organism,* Lactococcus garvieae*. This organism has been associated in contact with raw fish [9]. However, in our case the origin was not known. It did prompt an inpatient colonoscopy evaluation. There have been few case reports that suggest gastrointestinal risk factors in recent literature such as colonic diverticulosis, duodenal ulcers, and colonic polyps, but the evidence is poor [8, 9]. It is difficult to draw a conclusion that the patient's* Lactococcus garvieae* bacteremia was the result of the colonic pathology. However, there is more literature to support that* S*.* gallolyticus* subsp.* pasteurianus* bacteremia warrants colonic evaluation.

As there are no guidelines for managing patients with* S. gallolyticus* subsp.* pasteurianus* bacteremia, a colonoscopy should be considered as a part of the evaluation when no definitive source is identifiable. These premalignant lesions may progress to malignant colon cancers over the course of years and addressing these lesions earlier in their clinical course would prove beneficial to the patient in decreasing overall morbidity and mortality. Early detection of premalignant and malignant colonic lesions saves lives, and the discovery of such bacteremia should be investigated with a screening colonoscopy.

## Figures and Tables

**Table 1:** Previous and current classifications of *Streptococcus bovis* and their respective associations with colorectal cancer (CRC).

Previous nomenclature	Current nomenclature	Association with CRC
*S*. *bovis* biotype I	*S*. *gallolyticus* subsp. *gallolyticus*	++++

*S*. *bovis* biotype II/1	*S*. *infantarius* subsp. *infantarius*	++
	*S*. *lutetiensis*	++

*S*. *bovis* biotype II/2	*S*. *gallolyticus* subsp. *pasteurianus*	+
	*S*. *gallolyticus* subsp. *macedonicus*	−

## References

[1] Abdulamir A. S., Hafidh R. R., Abu Bakar F. (2011). The association of *Streptococcus bovis/gallolyticus* with colorectal tumors: the nature and the underlying mechanisms of its etiological role. *Journal of Experimental and Clinical Cancer Research*.

[2] Kahveci A., Ari E., Arikan H., Koc M., Tuglular S., Ozener C. (2010). *Streptococcus bovis* bacteremia related to colon adenoma in a chronic hemodialysis patient. *Hemodialysis International*.

[3] Abdulamir A. S., Hafidh R. R., Mahdi L. K., Al-jeboori T., Abubaker F. (2009). Investigation into the controversial association of Streptococcus gallolyticus with colorectal cancer and adenoma. *BMC Cancer*.

[4] Murinello A., Mendonca P., Ho C. (2006). Streptococcus gallolyticus bacteraemia associated with colonic adenomatous polyps. *Jornal Português de Gastrenterologia*.

[5] Corredoira J., Alonso M. P., Coira A., Varela J. (2008). Association between Streptococcus infantarius (formerly *S. bovis* II/1) bacteremia and noncolonic cancer. *Journal of Clinical Microbiology*.

[6] Boleij A., Van Gelder M. M. H. J., Swinkels D. W., Tjalsma H. (2011). Clinical importance of *Streptococcus gallolyticus* infection among colorectal cancer patients: systematic review and meta-analysis. *Clinical Infectious Diseases*.

[7] Romero B., Morosini M.-I., Loza E. (2011). Reidentification of *Streptococcus bovis* isolates causing bacteremia according to the new taxonomy criteria: still an issue?. *Journal of Clinical Microbiology*.

[8] Russo G., Iannetta M., D'Abramo A. (2012). Lactococcus garvieae endocarditis in a patient with colonic diverticulosis: first case report in Italy and review of the literature. *New Microbiologica*.

[9] Heras Cañas V., Pérez Ramirez M. D., Bermudez Jiménez F. (2015). *Lactococcus garvieae*endocarditis in a native valve identified by MALDI-TOF MS and PCR-based 16s rRNA in Spain: a case report. *New Microbes and New Infections*.

[10] Khan A. A. (2012). *Bacteria and Cancer*.

[11] Takamura N., Kenzaka T., Minami K., Matsumura M. (2014). Infective endocarditis caused by *Streptococcus gallolyticus* subspecies pasteurianus and colon cancer. *BMJ Case Reports*.

[12] Boleij A., Schaeps R. M. J., Tjalsma H. (2009). Association between *Streptococcus bovis* and colon cancer. *Journal of Clinical Microbiology*.

